# TMEM16F Aggravates Neuronal Loss by Mediating Microglial Phagocytosis of Neurons in a Rat Experimental Cerebral Ischemia and Reperfusion Model

**DOI:** 10.3389/fimmu.2020.01144

**Published:** 2020-07-07

**Authors:** Yijie Zhang, Haiying Li, Xiang Li, Jie Wu, Tao Xue, Jiang Wu, Haitao Shen, Xiang Li, Meifen Shen, Gang Chen

**Affiliations:** Brain and Nerve Research Laboratory, Department of Neurosurgery, The First Affiliated Hospital of Soochow University, Suzhou, China

**Keywords:** cerebral ischemia, TMEM16F, phagocytosis, neuronal cell death, microglia

## Abstract

Cerebral ischemia is a severe, acute condition, normally caused by cerebrovascular disease, and results in high rates of disability, and death. Phagoptosis is a newly recognized form of cell death caused by phagocytosis of viable cells, and has been reported to contribute to neuronal loss in brain tissue after ischemic stroke. Previous data indicated that exposure of phosphatidylserine to viable neurons could induce microglial phagocytosis of such neurons. Phosphatidylserine can be reversibly exposed to viable cells as a result of a calcium-activated phospholipid scramblase named TMEM16F. TMEM16F-mediated phospholipid scrambling on platelet membranes is critical for hemostasis and thrombosis, which plays an important role in Scott syndrome and has been confirmed by much research. However, few studies have investigated the association between TMEM16F and phagocytosis in ischemic stroke. In this study, a middle-cerebral-artery occlusion/reperfusion (MCAO/R) model was used in adult male Sprague-Dawley rats *in vivo*, and cultured neurons were exposed to oxygen-glucose deprivation/reoxygenation (OGD/R) to simulate cerebral ischemia-reperfusion (I/R) injury *in vitro*. We found that the protein level of TMEM16F was significantly increased at 12 h after I-R injury both *in vivo* and *in vitro*, and reversible phosphatidylserine exposure was confirmed in neurons undergoing I/R injury *in vitro*. Additionally, we constructed a LV-TMEM16F-RNAi transfection system to suppress the expression of TMEM16F during and after cerebral ischemia. As a result, TMEM16F knockdown alleviated motor function injury and decreased the microglial phagocytosis of viable neurons in the penumbra through inhibiting the “eat-me” signal phosphatidylserine. Our data indicate that reducing neuronal phosphatidylserine-exposure via deficiency of TMEM16F blocks phagocytosis of neurons and rescues stressed-but-still-viable neurons in the penumbra, which may contribute to reducing infarct volume and improving functional recovering.

## Introduction

Cerebral ischemia is a major cause of human neurological morbidity and mortality and is associated with a poor prognosis worldwide. It is caused by an interrupted blood supply to the brain and can result in severe neurodegeneration and vascular dementia in patients (1). According to the World Health Organization (WHO) data, the incidence of stroke in China is increasing at an annual rate of 8.7%. The annual death toll of stroke is over two million and about 85% of these patients died from ischemic stroke, which seriously threaten peoples' lives and affects their quality of life (2, 3).

Neurons in different ischemic regions experience different death patterns. Inflation, as a form of necrosis that is currently considered to be unprogrammed, often occurs in the ischemic core, where the reduction of blood flow is most pronounced. However, in areas of partial ischemia (the penumbra) and in areas around the infarct (peri-infarct), neurons stressed by ischemia, or its consequences are lost only after some delay, which usually in several programmed death modes such as neuronal apoptosis, PARP-1 dependent necrosis, and iron death, provides an opportunity for therapeutic interference hours or days after the stroke (4, 5).

Phagocytosis is normally secondary to the target cell dying by other means, such as apoptosis (6, 7). However, in viable phosphatidylserine (PS)-exposed cells, phagocytosis can directly cause cell death and such death is referred to as “primary phagocytosis,” with the defining characteristic that inhibition of phagocytosis can prevent cell death. Primary phagocytosis normally does not initiate cell death but rather executes cell death via phagocytosis, which may be induced by exposure of eat-me signals (such as PS) on viable target cells (4).

Recent data indicate that phagocytosis can execute the death of stressed-but-viable neurons during inflammation and neuropathology (8–10). Importantly, neurons in peri-infarct areas have been shown to expose PS in a reversible manner (11); these neurons can survive long-term if the phagocytosis is blocked. During the inflammation process, the PS exposed to neurons is bound by MFG-E8 (Milk fat globule EGF-like factor-8), which can induce phagocytosis via the microglial vitronectin receptor. Thus, blocking exposed PS, MFG-E8 or the vitronectin receptor can prevent neuronal loss and leave viable neurons without inhibiting inflammation. It has been reported that deficiency of MFG-E8 or MerTK (MER receptor tyrosine kinase, a receptor on the microglia) could prevent neuronal loss and improve physical function (10, 12). Therefore, our present study was designed to identify whether blocking PS exposure is enough to inhibit phagocytosis.

Calcium-activated phospholipid scramblase, also known as TMEM16F protein (13, 14), plays an important role in reversible PS-exposure. We therefore hypothesized that TMEM16F may contribute to the PS-exposure of viable neurons in the penumbra after transient cerebral ischemia, and thus promote phagocytosis. Our data showed that phagocytosis induced neuronal death after ischemia; knocking down the expression of TMEM16F could therefore prevent neuronal loss and mitigate long-term functional deficits.

## Materials and Methods

### Animals and Ethics

Adult male Sprague-Dawley (SD) rats weighing 300–350 g were purchased from the Animal Center of the Chinese Academy of Sciences (Shanghai, China). All experimental rats had adequate food and water, and they were housed in a quiet humidity- and temperature-controlled environment under a regular light/dark schedule (12/12 h light/dark cycle with humidity of 60 ± 5% and temperature of 22 ± 3°C). Primary neuronal cultures *in vitro* were prepared using 16–18-days-old pregnant SD rats. We strived to reduce the use of animals and relieved their pain as much as possible. The animal experimental protocols were approved by the Animal Care and Use Committee of Soochow University and performed in accordance with the National Institute of Health's guidelines (NIH Publication No. 8023, revised 2011).

### MCAO Model Establishment

Rats were subjected to 2 h right middle cerebral artery occlusion (MCAO) using a modified intraluminal filament technique that was first put forward and interpreted by Koizumi et al. (15) in rats. The rats were weighed, and then administered an intra-peritoneal injection of 4% chloral hydrate (0.1 ml/10 g i.p.) as an anesthetic agent (16, 17). After the anesthesia, the right common carotid artery (CCA), external carotid artery (ECA), and internal carotid artery (ICA) of rats were exposed through an incision in the middle on the neck. Subsequently, the proximal CCA and ECA were ligated, and an arterial clip was placed on the distal end of the CCA in order to block the blood flow to prevent bleeding when the filament was inserted. A filament with a diameter of 0.38 mm, whose tip was rounded by heating and coating with 0.01% poly-L-lysine, was inserted into the right CCA. The filament was advanced 18–20 mm further above the bifurcation until there was resistance, reaching, and occluding the ostium of the right middle cerebral artery (MCA). After that, the incision of the ICA was ligated, and the filament was secured in place for 2 h, after which the wound was closed, and the animals were allowed to awaken. Body temperature was maintained at 36.5–37.5°C using a heating pad during the procedure. Two hours post-occlusion, the filament was slowly withdrawn under anesthesia and animals were then returned to their cages for reperfusion (MCAO/R) (18, 19). Animals were assessed for functional impairment using the modified Bederson grading system to verify accurate occlusion of the middle cerebral artery when the rats were awakened (20, 21). Motor deficits were graded from 0 to 4. A score of 0 was given for no visible neurological deficits; a score of 1 was given for forelimb flexion; a score of 2 was given for contralateral weak forelimb grip (the operator places the animal on an absorbent pad and gently pulls the tail); a score of 3 was given for circling to the paretic side only when the tail was stimulated; and a score of 4 was given for spontaneous circling (20, 22, 23). Finally, animals with positive performances were included in our study.

### Cell Culture

Primary rat cortical neurons were obtained and cultured as described previously (24). Briefly, cortical neurons were prepared from embryonic-day-18 brains. Then, cortical neurons were digested with 0.25% trypsin-EDTA solution for 5 min at 37°C. The dissociated neurons were seeded on to six-well plates (Corning, USA) precoated with poly-D-lysine (Sigma, USA), and were cultured in Neurobasal medium containing 2% B-27, 0.5 mM of GlutaMAX, 50 U/ml of penicillin, and 50 U/ml of streptomycin (all from Invitrogen, Grand Island, NY, USA) under humidified air containing 5% CO_2_ at 37°C. The medium was renewed every 2 days until cell confluency was reached.

### Experiment Design

To verify the protein level of TMEM16F after ischemic attack (Experiment 1; Supplementary Figure 1A), rats under sham or MCAO/R surgeries were included. Animals experiencing MCAO/R were sacrificed at different intervals (6, 12, 24, 48, 72 h, or 7 d after MCAO/R). Then, brain tissue samples were obtained for analysis. We made a coronal cut at 3 and 9 mm from the front of the frontal lobe, taking a 6 mm thick brain tissue block. Regions from this section that corresponded to the ischemic core and penumbra were dissected. We then made a longitudinal cut (from top to bottom) ~2 mm from the sagittal suture through the right hemisphere. This was done to avoid mesial hemispheric structures, which are supplied primarily by the anterior cerebral artery. We then made a transverse diagonal cut at approximately at the “2 o'clock” position (as shown in Supplementary Figure 2) to separate the core (striatum and overlying cortex) from the penumbra (adjacent cortex) (25). The penumbra tissues surrounding the infarcted core of six rats from each group were extracted and frozen at −80°C until subsequent Western blot analysis. Additionally, we also confirmed the protein level of TMEM16F *in vitro*. Primary neuron cells were randomly and equally divided into eight groups, namely a control group and seven intervention groups including 2, 4, 6, 12, 24, 48, and 72 h after oxygen and oxygen-glucose deprivation reoxygenation (OGD/R). At the appropriate times after OGD/R for each group, cellular proteins were extracted for Western blot analysis.

To investigate the function of TMEM16F after transient cerebral ischemia *in vivo* (Experiment 2; Supplementary Figure 1B), specific lentivirus (LV-RNAi) against TMEM16F was applied to knock down TMEM16F. Rats were divided into the following four groups: sham group (sham), MCAO/R group, MCAO/R+ LV-negative control group (NC), and MCAO/R+ LV-TMEM16F-RNAi group (LV-RNAi). The MCAO/R surgery was performed 5 d after the transfection of LV-RNAi *in vivo*. At 12 h after MCAO/R, rats were sacrificed and the brain samples were collected for analysis. Rats in the neurobehavior groups were tested at 0-, 3-, 5-, 7-, and 14-days intervals after MCAO/R. To confirm the function of TMEM16F and its possible mechanism (Experiment 2; Supplementary Figure 1B), the following *in vitro* studies were conducted. Based on the interventions 96 h before OGD/R, the primary cultured neurons were divided into the following five groups: control group (Control), OGD/R group, OGD/R+ HitransG A reagent (HA, a reagent that could enhance transfection efficacy), OGD/R+HA+ LV-negative control group (NC), and OGD/R+ HA+LV-TMEM16F-RNAi group (LV-RNAi). At 12 h after OGD/R, cells were used for testing. In addition, at 12 and 24 h after OGD/R, cells that were not transfected with lentivirus were collected for flow cytometry analysis. Details of mortality and exclusion of experimental rats are shown in Supplementary Table 1.

### Establishing an Oxygen-Glucose Deprivation/Reoxygenation Model *in vitro*

Briefly, Neurobasal medium was replaced with DMEM (GIBCO, Carlsbad, CA, USA) and cells were transferred to a 5% CO_2_ and 95% N_2_ atmospheric incubator for 2 h at 37°C. After that, neurons were cultured in Neurobasal medium again and maintained in a 5% CO_2_ atmospheric incubator for the indicated time periods. Control groups were cultured in Neurobasal medium in a 5% CO_2_ atmospheric incubator for the same period. The pH of culture medium was maintained at 7.2 (26).

### Construction of LV-TMEM16F-RNAi System

#### In vivo

SD male rats (260–280 g body weight) were anesthetized with 4% chloral hydrate (0.1 ml/10 g) via an intra-peritoneal injection. Cortical injection of LV-TMEM16F-RNAi-GFP or LV-NC-GFP was carried out using a stereotaxic instrument. Each rat was subjected to three cortical injections as follows: AP +1.2 (site 1), −0.3 (site 2), −1.8 (site 3); ML +3.0; DV −3.0 from the skull. All of the target points were in the right hemisphere (i.e., ipsilateral to the MCAO). Two microliters of lentivirus suspension containing 9 × 10^8^ TU/ml were injected into each point at a rate of 0.2 μl/min (27, 28). The needle was slowly removed 5 min after completion of each injection. The rats were allowed 5 days of recovery before being subjected to the MCAO/R surgery.

#### In vitro

Cultured neurons were transfected with the LV-TMEM16F-RNAi-GFP or LV-NC-GFP combined with HitransG A reagent (HA), which could enhance transfection efficacy (GeneChem, China), according to the manufacturer's instructions. At 16 h after transfection, the medium was replaced and the neurons were incubated for another 4 days for further analysis.

### TTC Staining

The remaining rats were put to death by cutting off their cervical spines; their brains were quickly removed, and five 2 mm thick coronal slices were cut from each brain using razor blades. The slices were immersed in a physiological solution containing 2% (w/v) 2,3,5-triphenyltetrazolium hydrochloride (TTC) and incubated in a 37°C incubator for 15 min in the dark (29, 30). The infarcted tissue was verified by the complete loss of TTC staining, contrasting with the red-stained viable tissue. The area of infarction was determined by ImageJ software (Rawak Software Inc., Stuttgart, Germany).

### Western Blot Analysis

Western blot analysis was performed as in previous studies (31, 32). Briefly, the brain samples of rats or extracted neurons were mechanically lysed in a RIPA lysate buffer (Beyotime, China). The protein concentrations were measured with the bicinchoninic acid (BCA) method using a specific assay kit (Beyotime, China). The protein samples (30 μg/lane) were loaded onto a 10% SDS-polyacrylamide gel, separated, and then electrophoretically transferred to a polyvinylidene difluoride (PVDF) membrane (Millipore Corporation, USA). The membrane was blocked with 5% bovine serum albumin (BSA, BioSharp, China) at 25°C for 1 h. Next, the membrane was incubated with a primary antibody against TMEM16F (Cloud-Clone Corp., PAF813Hu01, 1:1,000 dilution) at 4°C overnight. The primary antibody against β-tubulin (Cell Signaling Technology, Danvers, USA) was diluted 1:5,000 and served as a loading control. Lastly, the membranes were incubated with a horseradish peroxidase-linked secondary antibody (goat anti-rabbit IgG-HRP, Cell Signaling Technology, Danvers, USA) for 2 h at 37°C and were washed three times with PBST (PBS + 0.1% Tween 20). Finally, the membrane was revealed with an enhanced chemiluminescence (ECL) kit (Beyotime Institute of Biotechnology) and the relative quantities of proteins were analyzed with ImageJ software (Rawak Software Inc., Stuttgart, Germany). The details of the resource identifiers for antibodies are shown in Supplementary Table 2.

### Immunofluorescent Analysis

The brain tissue samples were fixed in 4% paraformaldehyde, embedded in paraffin, and cut into 4 μm sections. The cultured neurons were fixed with 4% paraformaldehyde. Brain sections were treated with citrate antigen retrieval solution at 100°C followed by blocking with 1% BSA and incubated with primary antibodies at 4°C overnight and corresponding secondary antibodies at 25°C for another 1 h. Light was avoided throughout the whole experimental procedure. The titers of primary antibodies used in immunofluorescence were as follows: rabbit polyclonal anti-TMEM16F antibody (Cloud-clone Corp., PAF813Hu01, 1:100 dilution), mouse monoclonal anti-NeuN antibody (Abcam, ab 104,224, 1:200 dilution), goat monoclonal anti-Iba1 antibody (ab 5076, 1:200 dilution), mouse monoclonal anti-MAP2 antibody (ab11267, 1:200 dilution), and goat monoclonal anti-GFAP antibody (ab535,54,1:200 dilution). Secondary antibodies were purchased from Life Technologies and included the following: Alexa Fluor-488 donkey anti-rabbit IgG antibody (A21206), Alexa Fluor-488 donkey anti-goat IgG antibody (A11055), Alexa Fluor-555 donkey anti-mouse IgG antibody (A31570), and Alexa Fluor-555 donkey anti-goat IgG antibody (A21432). Normal rabbit IgG, normal mouse IgG, and normal goat IgG were used as negative controls for the immunofluorescence assay. Nuclei were stained with DAPI mounting medium. Finally, the sections and cells were observed by a fluorescence microscope (OLYMPUS BX50/BXFLA/DP70; Olympus Co., Tokyo, Japan). The details of the resource identifiers for antibodies are shown in Supplementary Table 2.

### pSIVA Binding and Flow Cytometry

#### Flow Cytometry

After OGD/R, cells (1 × 10^6^ cell per well in a six-well plate) were treated according to the manufacturer's protocol. Briefly, ~6 × 10^6^ cells were harvested from tissue culture plates and centrifuged at 1,500 rpm for 5 min at room temperature. Medium supernatant was removed, and cells were washed once in PBS. Cells were then resuspended in 1X binding buffer at a concentration of 1 × 10^6^–1 × 10^7^/ml. We transferred 100 μl of the resuspended cells (1 × 10^5^ to 1 × 10^6^ cells) to each vial that would be run, and both 5 μl of pSIVA-IANBD (Novus Biologicals, NBP2-29382) and 5 μl of propidium iodide (PI) were added. Samples were incubated at room temperature for 20 min in the dark, then 400 μl of 1X binding buffer was added to each tube, and the samples were analyzed by flow cytometry using a Beckman Coulter flow cytometer (Mississauga, Ontario).

#### pSIVA Immunofluorescent Analysis

pSIVA-IANBD was dialyzed into medium and applied to the neurons at 10–20 ul/ml concentration, according to the protocols. In this test, fluorescently tagged annexin binds to externalized phosphatidylserine exposed on cell membranes, allowing for monitoring changes that occur at different stages of apoptosis in living cells (33). After 15 min incubation, the cells were fixed and immunostained using MAP2 or TMEM16F antibody. Then, they were incubated with Alexa Fluor 555 conjugated anti-mouse antibody, as described above. In particular, the exposed PS binding with pSIVA did not need extra staining with secondary anti-body and could be observed directly using a green fluorescence filter set. Nuclei were stained with DAPI mounting medium. Cells were observed by a fluorescence microscope (OLYMPUS BX50/BXFLA/DP70; Olympus Co., Tokyo, Japan).

### Neurobehavioral Evaluation

At 24 h after reperfusion, rats were tested for behavioral impairment by a scoring system and subjected to the rotarod test (for motor function), the adhesive-removal test (for sensorimotor function), and the Morris Water maze test (for lateralized skilled forelimb reaching) (34).

#### Rotarod Test

An accelerating rotarod test was employed before and at 3, 5, 7, and 14 days after MCAO to measure the motor function of rats (35). The diameter of the rotarod spindle was 10 cm. The speed of the spindle was increased from 4 to 30 rpm in 60 s and 30 rpm was maintained for a maximum of 300 s. When the rats lost their balance and fell off the rotarod, it triggered the sensor and the time was recorded. Through separation by two panels to prevent the rats from disturbing each other, three rats were able to run at the same time. Before testing, each rat received three training sessions per day for three consecutive days and the last three trials served as the baseline. Then, all rats received a test trial on an accelerating rotarod at all testing days after MCAO/R.

#### Adhesive-Removal Test

The adhesive removal test was performed as described previously (34). Briefly, the rats were removed from their home cage and trained to be familiar with the testing environment. Then, two small pieces of adhesive-backed paper dots were glued to the wrist of each forelimb. The rats were then gently returned to their testing cages and they typically contacted and then removed each stimulus one at a time using their teeth. The time required to contact and remove both stimuli from each limb was recorded in five trials per day for 3 days separately. If the rats were able to contact the dots and remove those within 10 s at the end of training, then the rats were included in the experimental group. Then, all rats received a test trial at all testing days after MCAO/R.

#### Morris Water Maze Test

Briefly, we trained the rats in the Morris water maze for 3–6 days (three trials a day) before the formal test. The tests were performed on days 22–26 after MCAO/R in order to evaluate long-term neurobehavioral impairment (36–38). The arena was 50-cm high and 180 cm in diameter and was filled with water to a height of 30 cm at 20–22°C. The platform was placed ~2 cm below the water surface. The starting point for each rat changed every day. Each test continued until the rat found the platform and stood on it for 2 s, or until 60 s had elapsed. After each test, the rats were placed on the platform for 20 s. During testing for each rat, we recorded its swimming speed, latency to find the hidden platform, and the path length of the swimming path to reach the hidden platform (39).

#### Neurological Scores

At 24 h after MCAO/R, neurological tests were conducted on six rats per group to assess behavioral impairments. Behavioral performance was scored according to a previously published scoring system (20), which was a revised version of the Bederson score.

### Statistical Analysis

All data are expressed as the mean ± SEM. GraphPad Prism 7 (GraphPad Software San Diego, CA, USA) was used for statistical analysis. Differences between groups were determined with two-tailed Student's *t*-tests or Mann-Whitney *U*-tests for comparisons between two groups or with a one-way analysis of variance (ANOVA) followed by Tukey's *post-hoc* tests for comparisons of more than two groups. Statistical significance was determined as *P* < 0.05 unless otherwise stated.

## Results

### General Observations

No significant differences were found in the body temperature, body weight, heartbeat, or mean arterial blood pressure of rats in any of the MCAO/R experimental groups (Supplementary Figure 3) (40). No animals died in the sham group (0/12), and the mortality rate of the rats after MCAO/R induction was 18.10% (19/105).

### TMEM16F Protein Level Increases in Penumbra Neurons After Ischemic Injury

**Figure 1:**
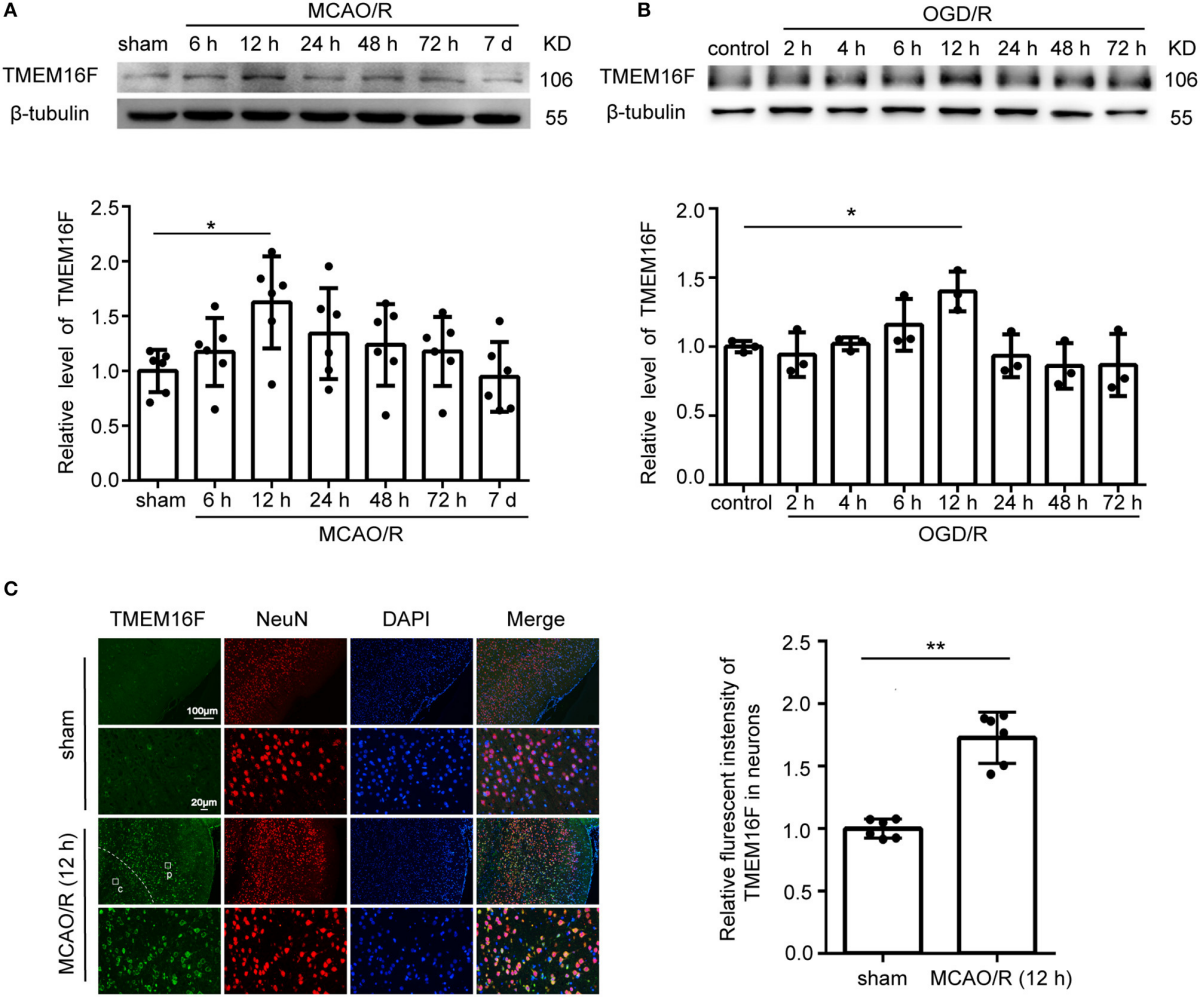
TMEM16F protein level increases after ischemic insult. **(A)** Western blot analysis and quantification of the protein level of TMEM16F in brain tissue around the penumbra after MCAO/R (*n* = 6, *P < 0.05). **(B)** Western blot analysis and quantification of the protein level of TMEM16F in cultured neurons after OGD/R treatment (*n* = 3, *P < 0.05). **(C)** Double immunofluorescence analysis was performed with TMEM16F antibodies (green) and a neuronal marker (NeuN, red) in brain sections. (p) represents the penumbra, and (c) represents the ischemic core. Nuclei were fluorescently labeled with DAPI (blue) (*n* = 6, **P < 0.01). In **(A–C)**, mean values for the sham group were normalized to 1.0. Data are presented as mean ± SEM. Differences were calculated with ordinary one-way ANOVA for **(A,B)** and Student's *t*-tests for **(C)**.

**Figure 2:**
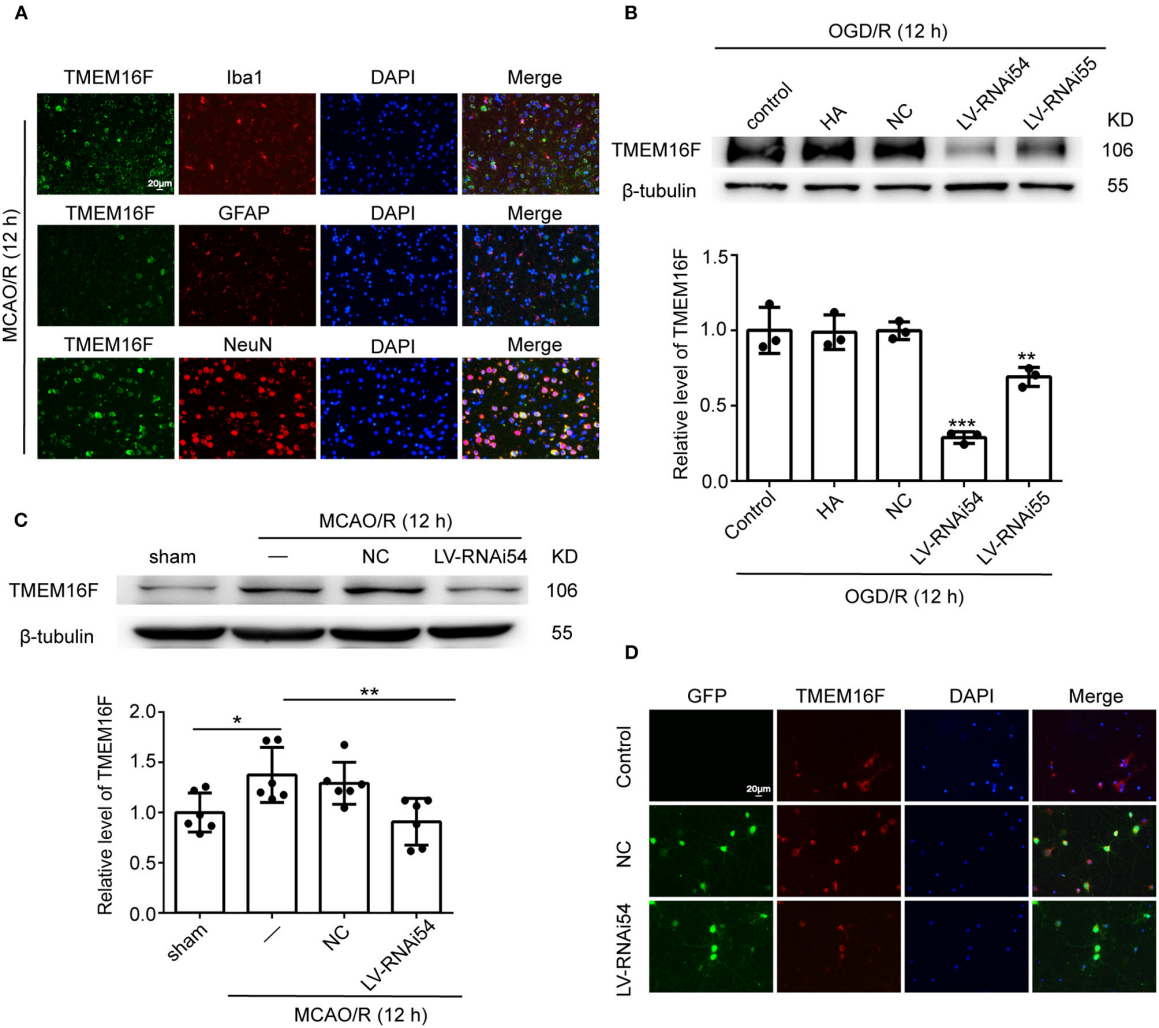
LV-TMEM16F-RNAi transfection downregulates TMEM16F in rat brain. **(A)** Double immunofluorescence analysis was performed with TMEM16F antibodies (green), a neuronal marker (NeuN, red), a microglia marker (Iba1, red), and an astrocyte marker (GFAP, red) in brain sections. Nuclei were fluorescently labeled with DAPI (blue) (scale bar = 20 μm). **(B)** Transfection efficiency of TMEM16F lentivirus (LV-RNAi) in cultured neurons. Quantifications of protein levels of TMEM16F in various groups are shown (*n* = 3, LV-RNAi54 vs. control/HA/NC; ***P < 0.001, LVRNAi55 vs. control/HA/NC, **P < 0.01). **(C)** Transfection efficiency of TMEM16F lentivirus (LV) in the rat brain. Quantifications of the protein level of TMEM16F in various groups are shown (*n* = 6, LV-RNAi54 vs. MCAO/R, **P < 0.01; MCAO/R/NC vs. sham, *P < 0.05). **(D)** Transfection efficiency of TMEM16F lentivirus (LV-RNAi) in cultured neurons was shown with immunofluorescence analysis. HitransG A (HA) is a reagent that could enhance transfection efficacy. In **(B,C)**, the mean values for the sham/control group were normalized to 1.0. Data are presented as mean ± SEM. Differences were calculated with ordinary one-way ANOVA.

To explore the expression of TMEM16F in brain tissue after MCAO/R, we detected the protein level of TMEM16F by western blotting *in vivo* (Figure 1A) and *in vitro* (Figure 1B). After MCAO/R modeling in rats, protein level of TMEM16F were significantly elevated at 6 h and peaked at 12 h (Figures 1A,B). In cultured primary neurons after OGD/R modeling, the protein level of TMEM16F was also significantly increased at 6 h and peaked by 12 h. Immunostaining of brain tissues after MCAO/R demonstrated that the protein level of TMEM16F was significantly elevated at 12 h compared with the sham group (Figure 1C). In addition, double immunofluorescence staining was performed to distinguish changes in cell type-specificity of TMEM16F expression after ischemia. The results showed that TMEM16F was mainly expressed in neurons under ischemic conditions and had a lower expression in microglia, whereas it was hardly detected in astrocytes (Figure 2A).

### TMEM16F Downregulation Reduces Motor Deficits After Ischemia

**Figure 3:**
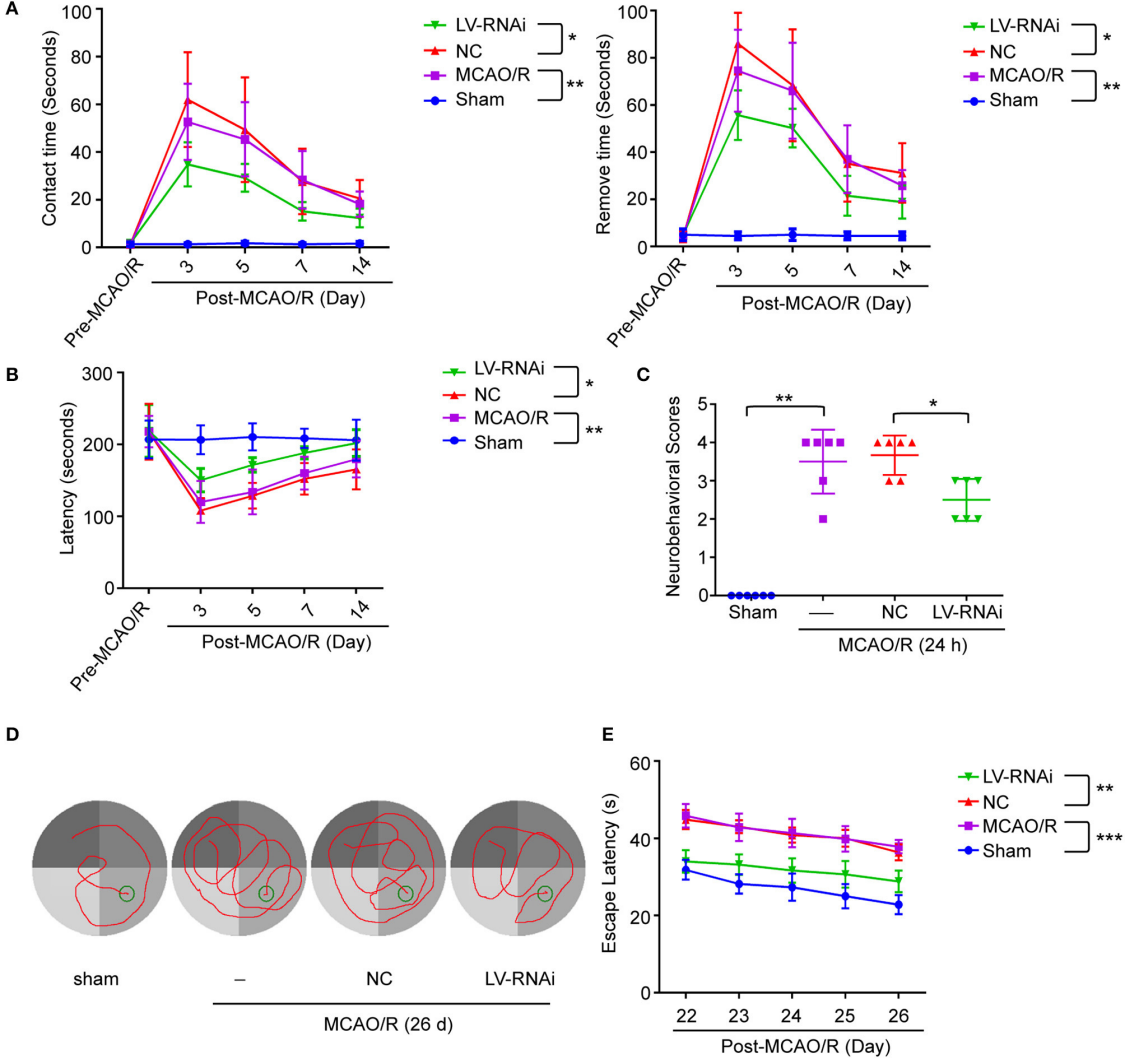
TMEM16F deficiency reduces motor deficits after focal cerebral ischemia. TMEM16F-deficiency rats show **(A)** shorter contact-time and removal-time in adhesive-removal tests at 3, 5, and 7 days after MCAO/R. (LV-RNAi vs. LV-NC at 3 d, *P* < 0.001; at 5 d, *P* < 0.01; at 7 d, *P* < 0.01) (*n* = 6, *P < 0.05, **P < 0.01). **(B)** longer latency time in rotarod test at 3,5,7, and 14 days. (LV-RNAi vs. LV-NC at 3 d, *P* < 0.001; at 5 d, *P* < 0.01; at 7 d, *P* < 0.01; at 14 d, *P* < 0.01) (*n* = 6, *P < 0.05, **P < 0.01). **(C)** lower scores in neurobehavior test (*n* = 6, *P < 0.05, **P < 0.01). **(D)** The typical swim path of rats in the Morris water maze test at 26 d after MCAO/R. **(E)** Time to reach the submerged platform in the water maze 22–26 d after MCAO/R. (LV-RNAi vs. LV-NC at all time points, *P* < 0.001) (*n* = 6, **P < 0.01, ***P < 0.001). Data are presented as mean ± SEM. Differences were calculated with two-way ANOVA for **(A,B,E)** and ordinary one-way ANOVA for **(C)**.

To further identify the role of TMEM16F in ischemic stroke, we adopted LV-RNAi against TMEM16F to decrease its expression. The interfering effects of LV-TMEM16F-RNAi were confirmed by western blotting both *in vivo* and *in vitro* (Figures 2B,C), and the transfection rate was observed using a microscopy *in vitro* experiment (Figure 2D). Two kinds of lentivirus targeted at TMEM16F were involved in the *in vitro* experiment, and we found LV-RNAi54 had better knockdown efficacy compared with LV-RNAi55 (Figure 2B). Thus, we chose LV-RNAi54 as the transfection reagent for further experiments. TMEM16F downregulation alleviated the sensory and motor deficits caused by MCAO/R, as suggested by the results of the adhesive-removal test (Figure 3A), rotarod test (Figure 3B), neurobehavioral scores (Figure 3C), and Morris water maze test (Figures 3D,E).

### Neurons Can Expose PS in a Reversible Manner After OGD/R

**Figure 4:**
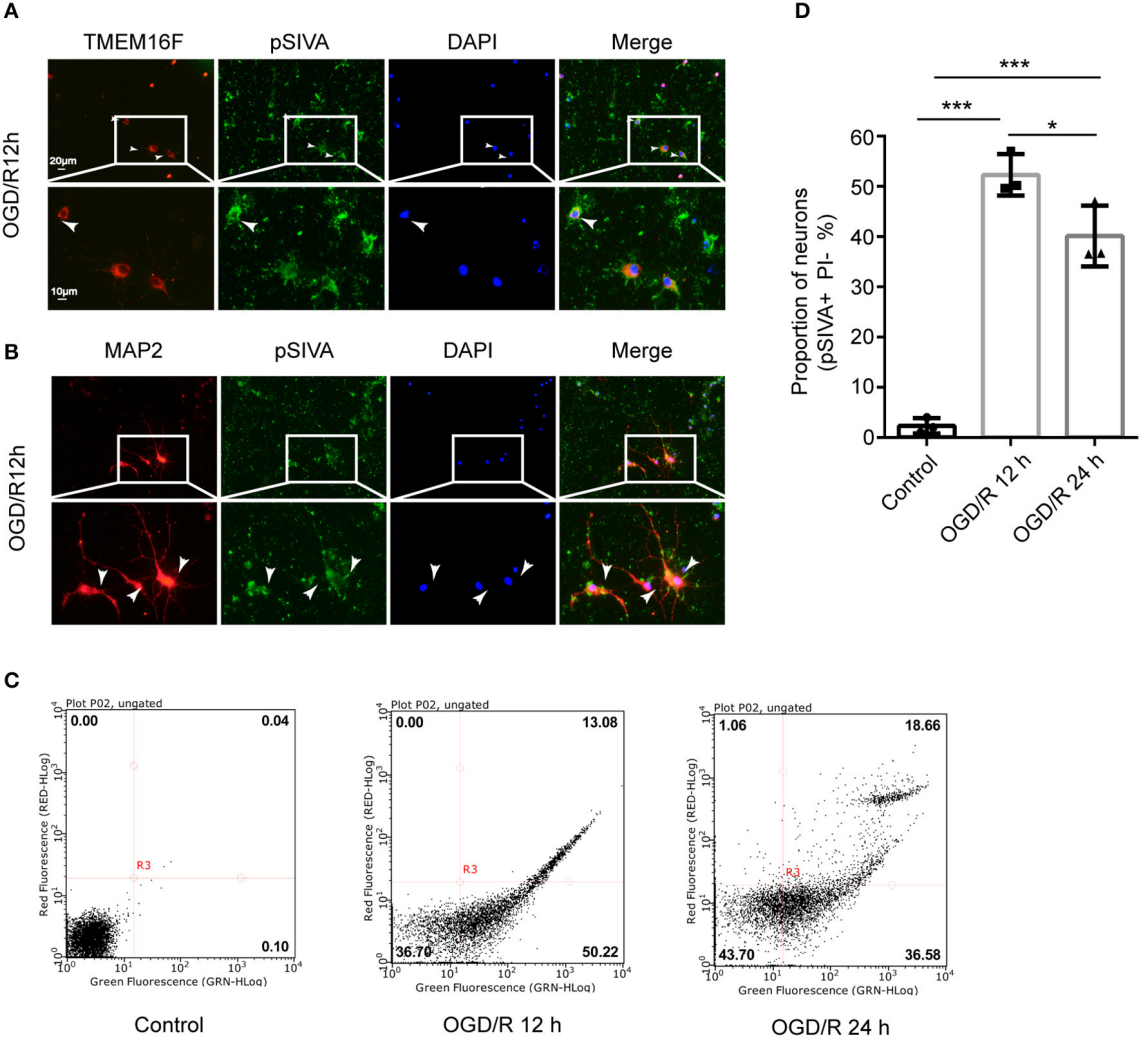
Neurons can expose PS in a reversible manner after OGD/R. **(A)** Cultured neurons were fluorescently labeled with TMEM16F antibodies (red) and an exposed PS marker (pSIVA, green). Nuclei were fluorescently labeled with DAPI (blue) (*n* = 3). **(B)** Cultured neurons were fluorescently labeled with neuronal antibodies (MAP2, red) and a PS marker (pSIVA, green). Nuclei were fluorescently labeled with DAPI (blue) (*n* = 3). **(C)** After OGD/R 12 and 24 h, PS-exposure were detected with pSIVA/PI double staining after incubation. **(D)** Proportion of PS-exposed neurons was compared between different groups (*n* = 3, OGD/R 12 h and OGD/R 24 h vs. control, ***P < 0.001; OGD/R 12 h vs. OGD/R 24 h, *P < 0.05). Data are presented as mean ± SEM. Differences were calculated with ordinary one-way ANOVA.

We found that the exposed PS was labeled by pSIVA-IANBD reagent with green fluorescence, distributed on the cell membrane of neurons, including the cell body as well as axons and dendrites, and co-located with TMEM16F at 12 h after OGD/R (Figures 4A,B). OGD/R caused extensive neuronal exposure of the eat-me signal PS, which is known to induce phagocytic uptake by microglia. However, at 24 h after OGD/R, the number of PS-exposing neurons (pSIVA+, PI–) was reduced significantly, and the proportion of healthy neurons (pSIVA–, PI–) increased (Figures 4C,D), compared with OGD/R 12 h. Additionally, there was no difference in the proportion of dead neurons between the two groups, which demonstrated that this PS exposure had been reversed to some extent in the absence of neuronal death or loss compared with OGD/R 12 h, indicating transient PS exposure on viable cells and that these neurons would live long-term without activated microglia joining in. Combined with the previous results, we speculate that knockdown of the expression of TMEM16F after ischemic injury probably protects the stressed-but-still-alive neurons in penumbra from undergoing phagocytosis by active microglia. Areas at the edge of the infarct (the penumbra) may have a chance to revert to healthy tissue when the PS exposure of viable neurons is inhibited.

Furthermore, in order to investigate whether PS exposure on the surface of neurons was reduced after knocking down TMEM16F, we collected cells after transfection with LV-TMEM16F-RNAi and performed flow cytometry, but the results were not satisfactory. We preferred the lentivirus for intervention because of its excellent and stable intervention effectiveness. However, in *in vitro* experiments, the transfection period of lentivirus was too long, inevitably affecting cellular status. Additionally, coupled with the effects of lentivirus transfection and OGD/R intervention, the neurons' status at this time was not good enough for flow cytometry analysis and could interfere with the experimental results. Short-term intervention methods (such as transfection with a designated plasmid) should be considered in future research. Other experimental methods should also be taken into account for measuring the PS exposure of attached neurons, as flow cytometry is not ideal.

### TMEM16F Deficiency Reduces Phagocytosis of Neurons After Focal Brain Ischemia

**Figure 5:**
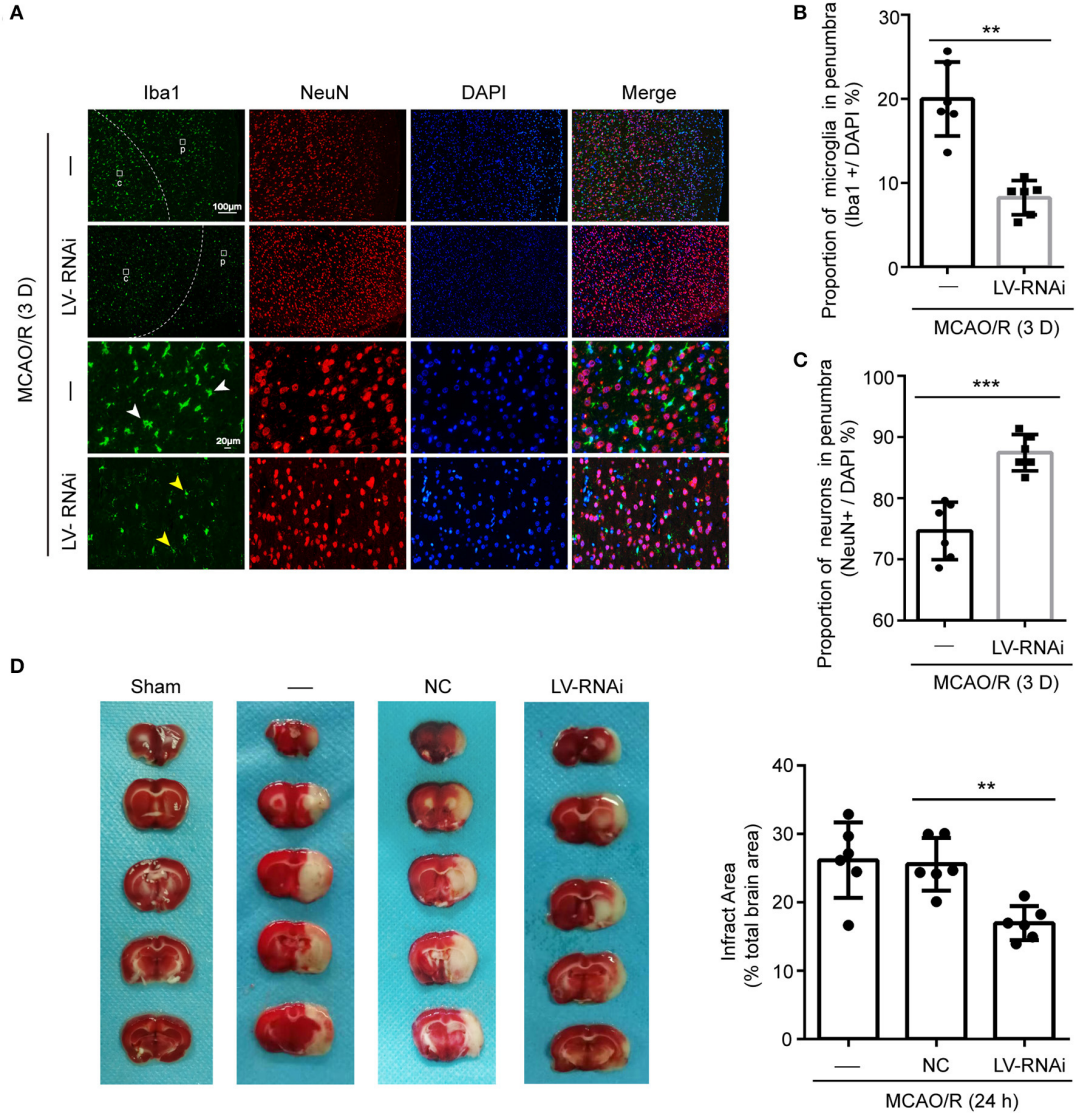
TMEM16F participates in phagocytosis of penumbra neurons after focal ischemia. **(A)** Macrophage/microglia activation in the penumbra of untreated and LV-RNAi transfected rats at 3 days after MCAO/R. Yellow arrowheads indicate ramified microglia, and white arrowheads indicate amoeboid microglia. (p) represents the penumbra and (c) represents the ischemic core. **(B)** Proportion of macrophages/microglia in the penumbra, indicating phagocytic activity: TMEM16F knockdown animals displayed decreased levels of phagocytic microglia at day 3 (*n* = 6, **P < 0.01). **(C)** Proportion of surviving neurons in the penumbra at 3 days after MCAO/R (*n* = 6, ***P < 0.001). **(D)** Triphenyltetrazolium chloride (TTC) staining showing the effects of TMEM16F intervention on neuronal loss after MCAO/R 24 h (*n* = 6, **P < 0.01). In **(B–D)**, data are presented as mean ± SEM. Differences were calculated with Student's *t*-tests for **(B,C)** and ordinary one-way ANOVA for **(D)**.

The numbers of microglia in the ischemic penumbra were determined by Iba-1-positive cell counts (12). A significant proportion of activated microglia had an amoeboid morphology which was characterized by darker cell bodies and thickened retracted processes, indicating a phagocytic phenotype (41). Additionally, the ramified/resting microglia were characterized by light round or oval cell bodies with fine symmetrical extended processes that were easy to distinguish (42). Few activated microglia were found at 1 d after ischemia, but a strong response was visible in the penumbra at 3 d, occurring in both the TMEM16F knockdown group and untreated animals (Figure 5A). This time-course of microglial recruitment and activation was similar to the time course of neuronal loss, as well as the recruitment of microglia in the core. However, at 3 d after ischemia there was a 11.8 ± 2.0% reduction in the number of active microglia in the TMEM16F knockdown group compared with the untreated group (Figure 5B), indicating that the phagocytic activity of microglia was reduced in TMEM16F-deficient animals. No differences were observed at other time points.

Furthermore, cell counts based on the neuronal nuclear antigen, NeuN, showed no loss of neurons at 1 d after ischemia, suggesting that neurons had not yet been phagocytosed. At 3 d, a few neurons had been lost in the penumbra (Figure 5C), but the proportion of neurons remaining in TMEM16F knockdown animals was significantly higher (untreated group: 74.7 ± 2.0%; TMEM16F knockdown: 87.5 ± 1.2%). Additionally, at the histological level, shown by TTC staining, deficiency of TMEM16F reduced the ischemic volume after MCAO/R (Figure 5D), indicating that suppressing the expression of TMEM16F could prevent stressed neurons from being phagocytosed by active microglia.

## Discussion

Microglia are the professional phagocytes of the brain and are able to rapidly phagocytose dead or dying neurons within hours (8, 43), which has been considered to be a beneficial process. However, microglia can also phagocytose stressed-but-viable neurons that reversibly expose to PS because of the calcium-activated scramblase TMEM16F (13, 44) or temporary lowering of ATP. Our data indicated that the deficiency of TMEM16F, which is required for reversible PS exposure, could prevent stressed-but-still alive neurons in a pre-infract area from being mistaken by microglia, thus blocking phagoptosis and further improving functional outcome after focal cerebral ischemia.

The affected area after cerebral ischemia can be divided into the core and the penumbra. It is generally assumed that in the central core of the ischemic area, which suffers from the highest level of ischemic injury, the cell death is oncotic/necrotic, whereas the penumbra experiences a lower level of ischemia in which neurons are functionally depressed but still viable at early time points (4). Besides, we should notice that the activation of microglia in the penumbra leads to a decrease in neuronal density over time, which means the hypoperfused penumbra may progress to infarct lesions and lead to more severe physical dysfunction (45). Therefore, timely rescue of neurons in the ischemic penumbra is of great significance for post-stroke treatment.

Phagoptosis, also called primary phagocytosis, is a recently recognized form of cell death caused by phagocytosis of viable cells that results in their destruction (46). The process of phagocytosis is normally initiated by the release of attractive signals from the target cell (referred to as “come-get-me”/“find-me” signals) leading to chemotaxis of a nearby macrophage (47). Upon reaching the target cell, the macrophage recognizes cell-surface signals on the target cell (“eat-me” signals), which then induce its uptake. The best characterized “eat-me” signal is the cell-surface exposure of phosphatidylserine PS (8, 10, 48, 49). In healthy cells, PS is found almost exclusively on the inner leaflet of the plasma membrane. However, PS can be exposed on the cell surface under certain conditions. PS exposure can occur as a result of: (i) apoptosis (when PS exposure occurs secondary to calcium elevation, ATP depletion, oxidative stress, or vesicle fusion); (ii) necrosis (due to plasma membrane rupture, calcium elevation or ATP depletion); (iii) calcium elevation (which stimulates the scramblase and inhibits the translocase); (iv) ATP depletion (which inhibits the translocase); (v) oxidative stress (which stimulates the scramblase and inhibits the translocase); or (vi) fusion of intracellular vesicles with plasma membrane (46, 50).

Studies have shown that PS-exposure not only occurs on the surface of cells as an early sign of cell death, but that it can also occur on the surface of viable cells in a reversible manner. It is provoked by exposure of “eat-me” signal PS on viable cells as a result of the activation of sublethal stimuli, potentially causing their phagocytosis when in the presence of phagocytes. For example, stressed but viable neurons can reversibly expose PS, but this only results in phagocytosis if activated microglia are present at the time of PS exposure (12).

**Figure 6:**
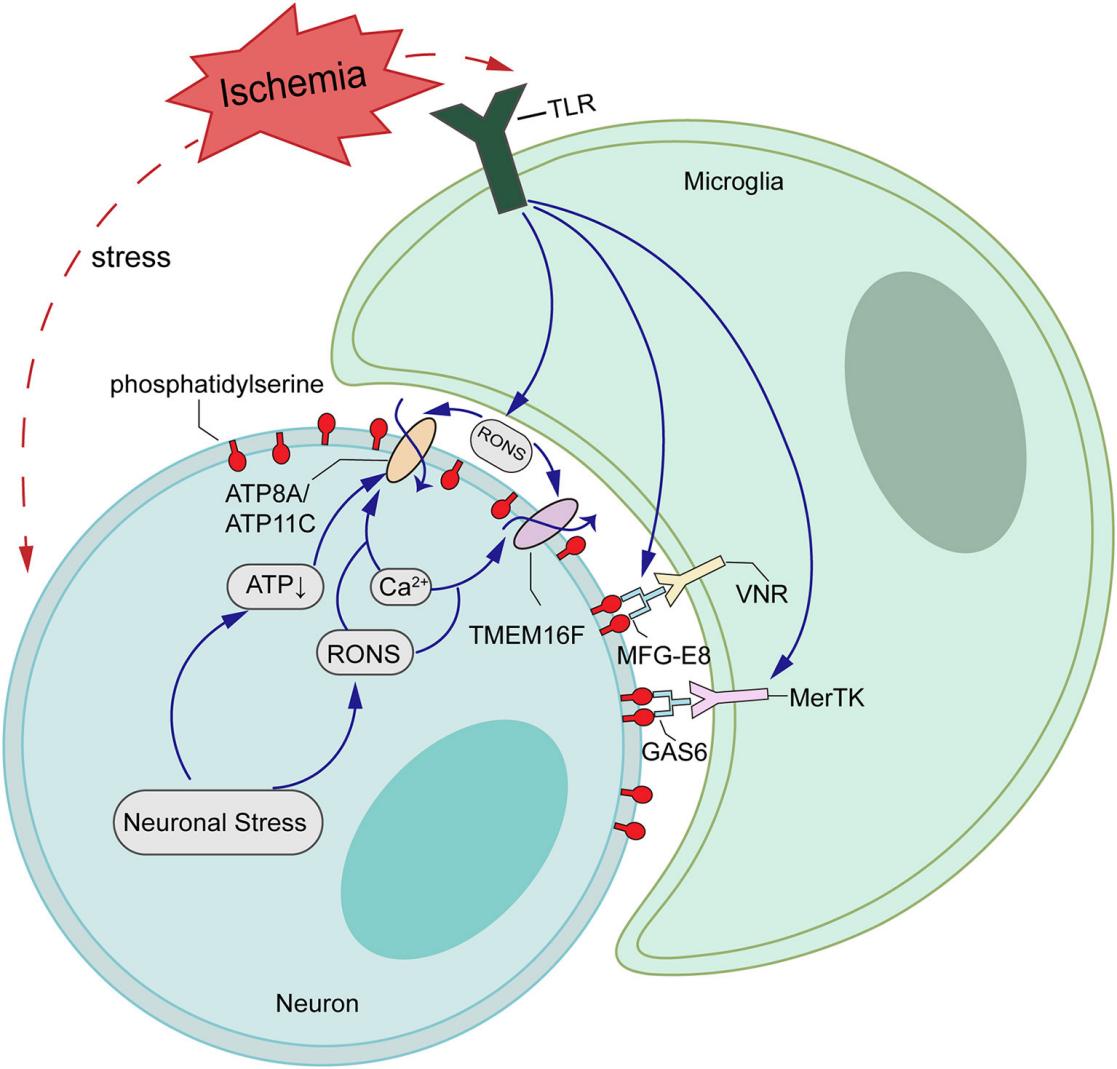
Schematic representations of potential mechanisms of TMEM16F actions in ischemia-reperfusion (I/R) injury. Ischemic stroke upregulates the expression of TMEM16F, which is activated by loaded intracellular Ca^2+^ and functions as a scramblase, inducing PS-exposure of stressed-but-alive neurons in the penumbra. Additionally, the flippases, such as ATP8A/ATP11C, are likely inactivated by the increasing Ca^2+^ or ATP depletion. When the stimuli are removed and cells return to a normal state, a decrease in intracellular Ca^2+^ inactivates TMEM16F, and the flippases restore the asymmetrical PS distribution.

In healthy cells that are not activated, PS is found exclusively on the inner leaflet of the plasma membrane, because the aminophospholipid translocase (also called “Flippases,” such as ATP11C and ATP8A) removes PS from the outer leaflet. However, a second enzyme, the phospholipid scramblase, can cause PS exposure by randomizing phospholipid distribution between the inner and outer leaflets (50, 51). In activated neurons, increased intracellular Ca^2+^ transiently activates TMEM16F to scramble phospholipids, probably inactivating flippase activity of ATP11C/ATP8A (Figure 6). When the stimulus is removed and cells return to a normal state, a decrease of intracellular Ca^2+^ inactivates TMEM16F, and the flippase restores the asymmetrical PS distribution (52, 53). We have shown here that OGD/R induces an excessive accumulation of intracellular Ca^2+^, which leads to the activation of TMEM16F, whose protein level reaches a peak at 12 h *in vitro*. Using pSIVA-IANBD reagent with green fluorescence to label exposed PS, we found that the exposed PS was distributed on the cell membrane of neurons and could co-locate with TMEM16F. Then we collected the cells at 12 and 24 h after OGD/R for flow cytometry analysis. Compared with 12 h reperfusion, the proportion of PS-exposed neurons was significantly decreased and that of healthy cells was increased after 24 h reperfusion without increasing cell death, suggesting a transient PS exposure on stressed-but-viable cells. In this study, microglia were not co-cultured with neurons, so we speculate that when activated microglia do not appear, stressed neurons can return to a normal state and survive longer.

Accordingly, other studies have shown that viable neurons in the postischemic brain can be labeled by annexin V, indicating that PS exposure is reversible at 4 h after transient ischemia. At the same time, the neuronal marker MAP2 is co-localized with annexin V staining, which means the neuronal structure still remains intact. However, by day 1, annexin V-positive cells had a neuronlike morphology, but lost MAP2 staining, suggesting that these cells had already suffered irreversible ischemic injury (11). These neurons can stay alive if activated microglia do not appear (54). We can infer from this that there may be a critical time point between 4 and 24 h at which neurons could not maintain structural integrity anymore and eventually move toward apoptosis. We found that the expression of TMEM16F, a Ca^2+^-activated phospholipid scramblase, was significantly elevated at 6 h after transient focal ischemia in rats and peaked at 12 h. At this point the scramblase was highly activated and probably increased the exposure of PS, as well as the risk of being phagocytosed.

Microglia also play an important role in this process. PS-exposed neurons are recognized either via the opsonin MFG-E8 and vitronectin receptors on microglia (10) or by the opsonin Gas6 and MerTK (Mer receptor tyrosine kinase) receptors on microglia (8, 9, 55). It appears that inflammatory activation of microglia impairs their ability to discriminate between apoptotic and viable neurons for phagocytosis, resulting in phagoptosis during inflammation (56). Microglia fail to recognize cells that should be removed and phagocytize transiently exposed PS neurons via the microglial vitronectin receptor MFG-E8, leading to an increase in the infarct size. A study had reported that transient brain ischemia caused a delayed neuronal loss, accompanied by microglial phagocytosis of neurons, which was prevented by genetic inactivation of either MFG-E8 or MerTK (12). Only a few activated microglia were found at 1 d after ischemia in our study, but a strong response was visible in the penumbra at 3 d, indicating that the phagocytosis of neurons was delayed by at least 72 h, which is consistent with previous research (41). We also found the proportion of Iba1+ cells were significantly decreased in penumbra after knocking down TMEM16F. In accordance with phagocytosis of viable neurons, deficiency in TMEM16F may protect neurons from phagocytic uptake and lead to a reduction in the infarct size after transient cerebral ischemia *in vivo*.

It has been reported that expression of TMEM16F is significantly higher in macrophages, where it supports phagocytic activity and cell death (57). Recent studies find that TMEM16F plays an important role in regulating spinal microglia function in neuropathic pain states (58) and spinal cord injury (59). Likely due to a different disease model and the microglia accounting for only 20% of brain tissue, TMEM16F was mainly expressed on the neuronal membrane instead of the microglia in our study after MCAO/R. Further studies are needed to identify whether TMEM16F deficiency also diminishes the branch motility and phagocytic capacity of microglia and contributes to viable neuronal survival in the penumbra after transient cerebral ischemia.

There were some technical limitations to the present study. First, we only used adult male rats to establish the MCAO/R model *in vivo*, so there may be a problem of gender bias. *In vitro*, we chose primary neurons to incubate and used a conditional neuronal basal medium, without mixing microglia, so the direct neuron-microglia contact could not be investigated. Additionally, our study explored only the role of TMEM16F after MCAO/R and, in pathological conditions, the downstream factors PS-binding protein MFG-E8 and vitronectin receptors such as MerTK and the relationship between them were not involved. Flippases such as ATP8A/ATP11C probably also contributed to reversible PS exposure cooperated with scramblase TMEM16F. Therefore, the cooperation between scramblase and flippases should be considered. Furthermore, there may be a TMEM16F-MFG-E8 and MerTK pathway in phagocytosis of stressed-but-viable neurons, which needs further research.

In summary, our study revealed that TMEM16F could be upregulated after transient cerebral ischemia and increase phagocytosis by microglia. Stressed neurons could expose PS in a reversible manner; deficiency of this phospholipid scramblase may protect stressed-but-viable neurons from being phagocytosed by activated microglia in the penumbra and impede the expansion of the infarct area. In the ischemic penumbra in patients, neuronal loss was detected several days or months post-stroke (60–62), which was consistent with our data. This important time course between the increase of neuronal PS exposure and the initiation of phagocytosis by activated microglia should be considered and may provide potential therapeutic targets.

## Data Availability Statement

All datasets generated for this study are included in the article/Supplementary Material.

## Ethics Statement

The animal study was reviewed and approved by the Animal Care and Use Committee of Soochow University.

## Author Contributions

YZ, HL, and XL (3rd Author) designed the study, collected and analyzed data, and wrote the manuscript. JW, TX, and JW collected and analyzed data and revised the manuscript. MS and GC supervised the experiments. HS and XL (8th Author) contributed to the study design and revised the manuscript. All authors gave final approval of the manuscript.

## Conflict of Interest

The authors declare that the research was conducted in the absence of any commercial or financial relationships that could be construed as a potential conflict of interest.
