# Supplementary material for: TMEM16F Aggravates Neuronal Loss by Mediating Microglial Phagocytosis of Neurons in a Rat Experimental Cerebral Ischemia and Reperfusion Model
Source: Front Immunol. 2020 Jul 7;11:1144. doi: 10.3389/fimmu.2020.01144 (PMC7359929; doi:10.3389/fimmu.2020.01144)
Supplement: Supplementary file 1 [file Table_1.docx]

Supplementary Material

# Supplementary Table

**Supplementary Table.1 Rat mortality and exclusion.**

| Group | Mortality Rate | Excluded |
| --- | --- | --- |
| Experiment 1 |  |  |
| Sham | 0% (0/6) | 0 |
| MCAO/R (6h,12h,24h,48h,72h,7d) | 20.37% (11/54) | 7 |
| Experiment 2 |  |  |
| Sham | 0% (0/6) |  |
| MCAO/R(12h) | 12.50% (2/10) | 2 |
| MCAO/R(12h)+ NC | 11.11% (1/9) | 2 |
| MCAO/R(12h)+ LV-RNAi | 10% (1/10) | 3 |
| MCAO/R(72h) | 18.18% (2/11) | 3 |
| MCAO/R(72h)+ LV-RNAi | 18.18% (2/11) | 3 |
| Total | 16.24% (19/117) | 20 |
| Sham | 0% (0/12) | 0 |
| MCAO/R | 18.10% (19/105) | 20 |
