# Supplementary material for: TMEM16F Aggravates Neuronal Loss by Mediating Microglial Phagocytosis of Neurons in a Rat Experimental Cerebral Ischemia and Reperfusion Model
Source: Front Immunol. 2020 Jul 7;11:1144. doi: 10.3389/fimmu.2020.01144 (PMC7359929; doi:10.3389/fimmu.2020.01144)
Supplement: Supplementary file 2 [file Table_2.DOCX]

Supplementary Material

# Supplementary Table

**Supplementary Table.2 Resource Identifiers for Antibodies**

| Antibody ID | — | AB_10711040 | AB_2224402 | AB_297885 | AB_880202 |
| --- | --- | --- | --- | --- | --- |
| Antibody name | TMEM16F antibody | Anti-NeuN Antibody | Anti-Iba1 antibody | Anti-MAP2 antibody | Anti-GFAP  Antibody |
| Target  antigen | human,  mouse, rat, pig | human, mouse, rat, pig, human, cow, porcine, bovine | rat, rabbit, pig, cow,dog, human, common marmoset | mouse, rat, chicken, cow, human, quail | human, mouse, rat |
| Vendor | Cloud-Clone Corp. | Abcam | Abcam | Abcam | Abcam |
| Cat number | PAF813Hu01 | ab104224 | ab5076 | ab11267 | ab53554 |
| Proper  citation | Cloud-Clone Corp.Cat# PAF813Hu01 | Abcam Cat# ab104224, RRID:AB_10711040 | Abcam Cat# ab5076, RRID:AB_2224402 | Abcam Cat# ab11267, RRID:AB_297885 | Abcam Cat# ab53554, RRID:AB_880202 |
| Reference | - | 17 | 63 | 13 | 28 |
| Clonality | polyclonal  antibody | monoclonal antibody | polyclonal antibody | monoclonal antibody | polyclonal antibody |
| Clone ID | - | - | - | Clone HM-2 | - |
| Host organism | Rabbit | Mouse | Goat | Mouse | Goat |
| Comments | WB; IHC; IF ICC; IP | WB, IHC-P, IF/ICC, IHC-FoFr, IHC-Fr | Electron Microscopy, IHC-P, WB, IHC-FrFl, ICC, ICC/IF | ICC, IHC-Fr, ICC/IF, IHC-FrFl, WB, IHC-P, IHC-FoFr | IHC-FrFl, WB, IHC-P, IHC-Fr, IHC-FoFr, ICC/IF |
| Application  notes | WB: 1:500-1:2000  IF: 1:100 | WB: 1:1000-2000  IF: 1:200 | WB: 1:1000  IF: 1:200 | WB: 1:1000  IF: 1:200 | WB: 1:500-2000  IF: 1:200 |
