# Supplementary material for: TMEM16F Aggravates Neuronal Loss by Mediating Microglial Phagocytosis of Neurons in a Rat Experimental Cerebral Ischemia and Reperfusion Model
Source: Front Immunol. 2020 Jul 7;11:1144. doi: 10.3389/fimmu.2020.01144 (PMC7359929; doi:10.3389/fimmu.2020.01144)
Supplement: Supplementary file 3 [file Data_Sheet_1.DOCX]

Supplementary Material

**Supplementary Figure 1.** **Experimental design.**


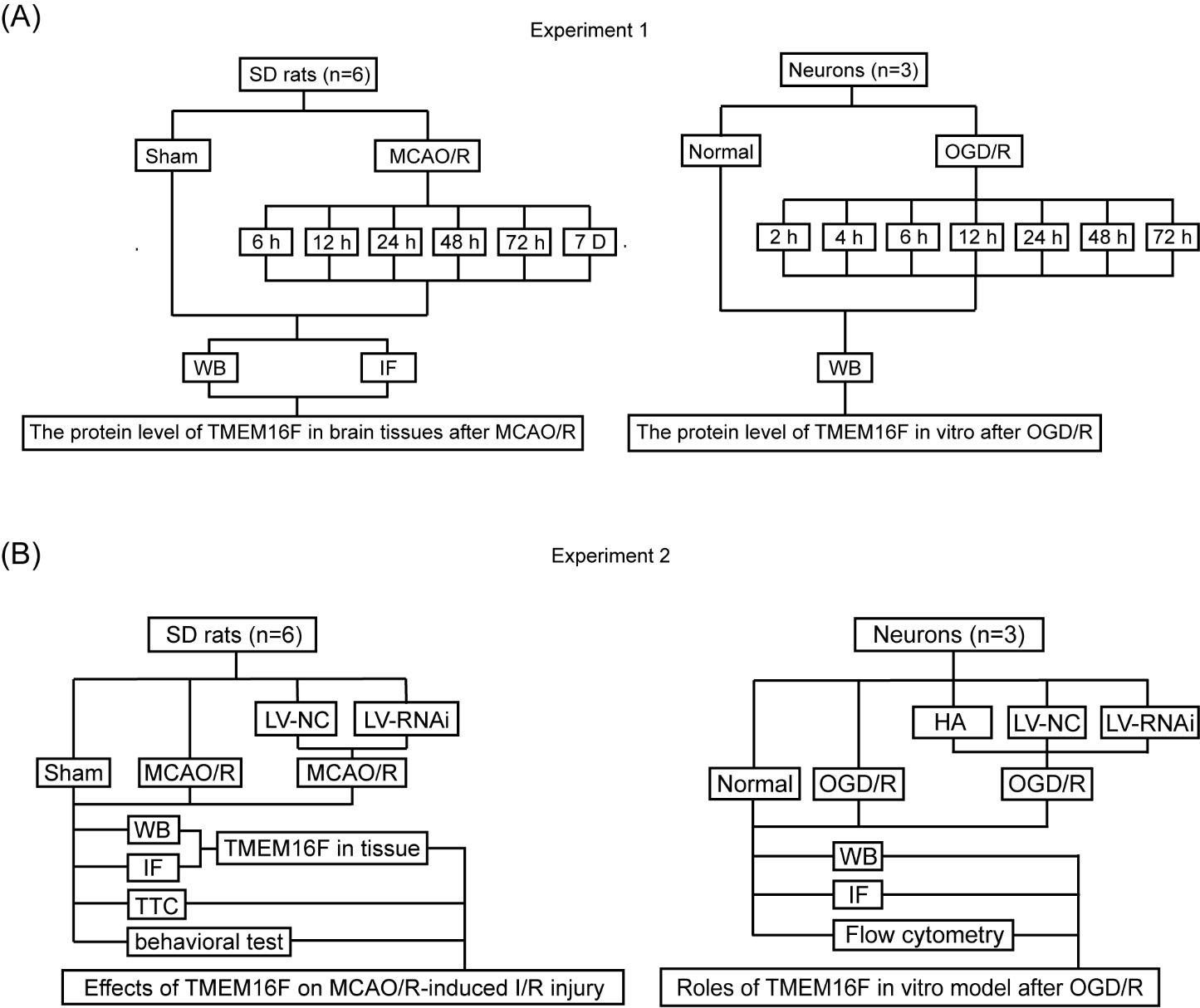


**Supplementary Figure 1.** **Experimental design.**

**(A)** Experimental design displaying the change of TMEM16F proteins at different time points after MCAO/R both *in vivo* and *in vitro*. **(B)** Effects of TMEM16F on MCAO/R-induced I/R injury in rats after the indicated interventions both *in vivo* and *in vitro*.
