# Supplementary material for: TMEM16F Aggravates Neuronal Loss by Mediating Microglial Phagocytosis of Neurons in a Rat Experimental Cerebral Ischemia and Reperfusion Model
Source: Front Immunol. 2020 Jul 7;11:1144. doi: 10.3389/fimmu.2020.01144 (PMC7359929; doi:10.3389/fimmu.2020.01144)
Supplement: Supplementary file 4 [file Data_Sheet_2.DOCX]

Supplementary Material

**Supplementary Figure 2.** **Separation of ischemic core and penumbra**


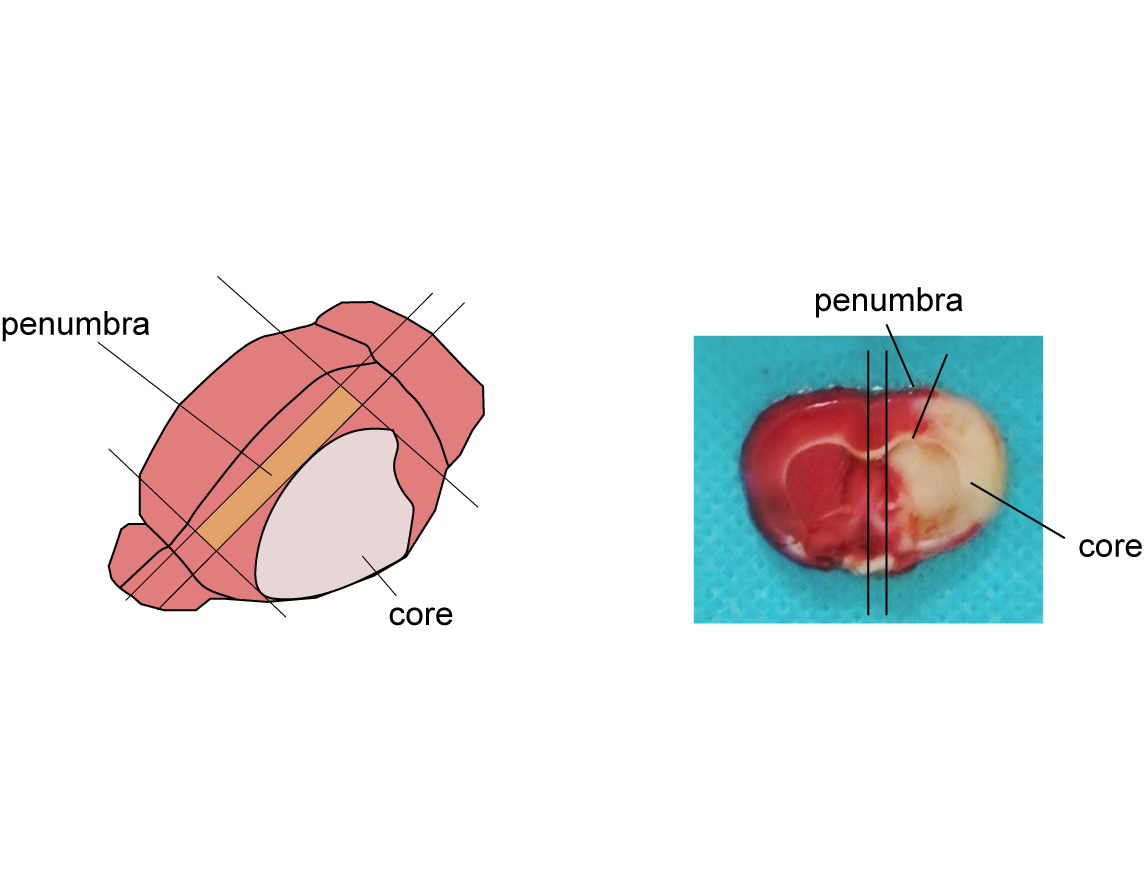


**Supplementary Figure 2. Separation of penumbra.**

We made a coronal cut at 3mm and 9mm from the front of the frontal lobe, took a 6mm thick brain tissue block. Regions from this section that corresponded to the ischemic core and penumbra were dissected. Then made a longitudinal cut (from top to bottom) approximately 2 mm from the sagittal suture through right hemisphere. This was done to avoid mesial hemispheric structures, which are supplied primarily by the anterior cerebral artery. We then made a transverse diagonal cut at approximately the “2 o’clock” position to separate the core (striatum and overlying cortex) from the penumbra (adjacent cortex).
