# Supplementary material for: TMEM16F Aggravates Neuronal Loss by Mediating Microglial Phagocytosis of Neurons in a Rat Experimental Cerebral Ischemia and Reperfusion Model
Source: Front Immunol. 2020 Jul 7;11:1144. doi: 10.3389/fimmu.2020.01144 (PMC7359929; doi:10.3389/fimmu.2020.01144)
Supplement: Supplementary file 5 [file Data_Sheet_3.DOCX]

Supplementary Material

**Supplementary Figure 3.** **General observations of rats**


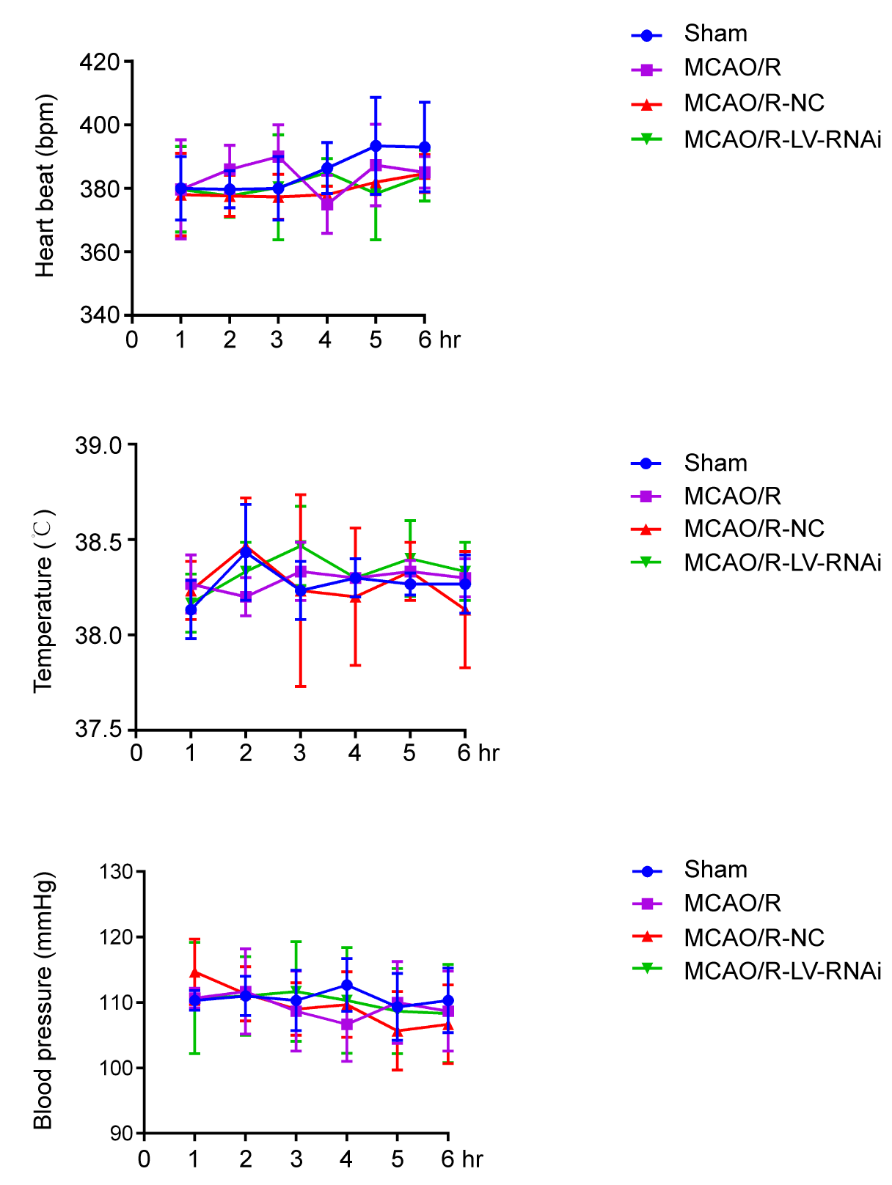


**Supplementary Figure 3.** Heart rate, blood pressure and body temperature were monitored within 6 hours after MCAO/R onset and were shown above. And there was not significant change among each group.
